# Association of Medication Intensity and Stages of Airflow Limitation With the Risk of Hospitalization or Death in Patients With Heart Failure and Chronic Obstructive Pulmonary Disease

**DOI:** 10.1001/jamanetworkopen.2018.5489

**Published:** 2018-12-14

**Authors:** Claire A. Lawson, Mamas A. Mamas, Peter W. Jones, Lucy Teece, Gerry McCann, Kamlesh Khunti, Umesh T. Kadam

**Affiliations:** 1 Leicester Diabetes Centre, University of Leicester, Leicester, United Kingdom; 2 Keele Cardiovascular Research Group, Centre for Prognosis Research, Institute of Primary Care and Health Sciences, Keel University, Stoke-on-Trent, United Kingdom; 3 Faculty of Medicine and Health Sciences, Keele University, Stoke-on-Trent, United Kingdom; 4 Department of Cardiovascular Sciences, University of Leicester, Leicester, United Kingdom; 5 National Institute for Health Research Biomedical Research Centre, Glenfield Hospital, Leicester, United Kingdom; 6 Department of Health Sciences, University of Leicester, Leicester, United Kingdom

## Abstract

**Importance:**

In heart failure (HF), chronic obstructive pulmonary disease (COPD) increases the risk of poor outcomes, but the effect of COPD severity is unknown. This information is important for early intervention tailored to the highest-risk groups.

**Objectives:**

To determine the associations between COPD medication intensity or stage of airflow limitation and the risk of hospitalization or death in patients with HF.

**Design, Setting, and Participants:**

This UK population-based, nested case-control study with risk-set sampling used the Clinical Practice Research Datalink linked to Hospital Episode Statistics between January 1, 2002, to January 1, 2014. Participants included patients aged 40 years and older with a new diagnosis of HF in their family practice clinical record. Data analysis was conducted from 2017 to 2018.

**Exposures:**

In patients with HF, those with COPD were compared with those without it. International COPD (Global Initiative for Chronic Obstructive Lung Disease [GOLD]) guidelines were used to stratify patients with COPD by 7 medication intensity levels and 4 airflow limitation severity stages using automatically recorded prescriptions and routinely requested forced expiratory volume in 1 second (FEV_1_) data.

**Main Outcomes and Measures:**

First all-cause admission or all-cause death.

**Results:**

There were 50 114 patients with new HF (median age, 79 years [interquartile range, 71-85 years]; 46% women) during the study period. In patients with HF, COPD (18 478 [13.8%]) was significantly associated with increased mortality (adjusted odds ratio [AOR], 1.31; 95% CI, 1.26-1.36) and hospitalization (AOR, 1.33; 95% CI, 1.26-1.39). The 3 most severe medication intensity levels showed significantly increasing mortality associations from full inhaler therapy (AOR, 1.17; 95% CI, 1.06-1.29) to oral corticosteroids (AOR, 1.69; 95% CI, 1.57-1.81) to oxygen therapy (AOR, 2.82; 95% CI, 2.42-3.28). The respective estimates for hospitalization were AORs of 1.17 (95% CI, 1.03-1.33), 1.75 (95% CI, 1.59-1.92), and 2.84 (95% CI, 1.22-3.63). Availability of spirometry data was limited but showed that increasing airflow limitation was associated with increased risk of mortality, with the following AORs: FEV_1 _80% or more, 1.63 (95% CI, 1.42-1.87); FEV_1_ 50% to 79%, 1.69 (95% CI, 1.56-1.83); FEV_1_ 30% to 49%, 2.21 (95% CI, 2.01-2.42); FEV_1_ less than 30%, 2.93 (95% CI, 2.49-3.43). The strength of associations between FEV_1_ and hospitalization risk were similar among stages ranging from FEV_1_ 80% or more (AOR, 1.48; 95% CI, 1.31-1.68) to FEV_1_ less than 30% (AOR, 1.73; 95% CI, 1.40-2.12).

**Conclusions and Relevance:**

In the UK HF community setting, increasing COPD severity was associated with increasing risk of mortality and hospitalization. Prescribed COPD medication intensity and airflow limitation provide the basis for targeting high-risk groups.

## Key Points

**Question:**

Who are the highest risk heart failure (HF) patients with chronic obstructive pulmonary disease (COPD)?

**Findings:**

In this nested case-control study of more than 50 000 UK patients with HF, COPD increased the risk of hospital admission and death by more than a third, but increased risk was specific to patients receiving the most intense COPD medication regimens: triple inhaler therapy, prescribed oral corticosteroids, or oxygen therapy. Spirometry recording was limited in the community setting was restricted to more severe patients with HF and COPD, and for these patients the risk of mortality significantly increased alongside increasing airway limitation.

**Meaning:**

Optimal care of patients with HF and COPD requires accurate diagnosis and targeting of severe COPD markers to prevent admissions and death.

## Teaser

This UK population-based nested case-control study assesses the associations between chronic obstructive pulmonary disease (COPD) medication intensity or stage of airflow limitation and the risk of hospitalization or death in patients with heart failure.

## Introduction

Heart failure (HF) and chronic obstructive pulmonary disease (COPD) are common diseases of aging and leading causes of morbidity and mortality worldwide.^1^ Old age, as well as shared risk factors and pathophysiology,^2^ means that these 2 diseases are often experienced together, with each influencing the clinical course of the other.^3^ Prevalence of COPD affects approximately a third of patients with HF^4^ and studies have consistently shown COPD to be associated with higher mortality for patients with HF. These studies were mostly conducted in small HF cohorts in specialist care populations or following discharge from hospital, with some evidence suggesting that the risk of COPD-associated death in HF might differ according to COPD severity.^5,6,7,8,9,10^ However, there is limited evidence on the impact of COPD on hospitalizations^11^ or on patients with HF in the community setting.^12^ Whether any elevated risk of hospitalization or death associated with COPD in community patients with HF differs according to clinical severity is unknown. This is important for identifying individuals with the greatest potential for treatment optimization of both conditions.

In a national cohort of patients with newly diagnosed HF, the current study investigated whether the associations between COPD and subsequent risk of hospitalization and death were significantly stratified by 2 severity measures of COPD: intensity of prescribed medications and routinely measured airflow limitation.

## Methods

### Study Population

We used the Clinical Practice Research Datalink (CPRD),^13^ an internationally used database of anonymized records from a representative sample of the UK general population,^14^ linked to the hospital episode statistics database of all admissions in the United Kingdom. The CPRD includes detailed patient demographic and clinical information and linkage to hospital episode statistics that are available for approximately 60% of the CPRD population. The CPRD has ethical approval from the Health Research Council to supply and link anonymized data from consenting family practices. Individual patient consent is not required but patients can opt out if they wish. The Independent Scientific Advisory Committee provided permission for this study. Data was reported following the Strengthening the Reporting of Observational Studies in Epidemiology (STROBE) reporting guideline.

From the database, the study included all patients aged 40 years or older with a new HF diagnosis in their clinical record between January 1, 2002, and March 1, 2012, and who had at least 3 years of CPRD-approved clinical data prior to their study entry. A clinically validated HF code set^15^ as well as additional HF-specific codes were used (eTable 1 in the Supplement). Patients were followed up until the first of the following: their date of transfer out of practice, their index outcome event, or January 1, 2014, which was the end of study (eFigure in the Supplement).

### Selection of Cases and Matched Controls

We conducted 2 separate nested case-control studies within the HF cohort for the outcomes of all-cause mortality and first all-cause first hospitalization after HF diagnosis. A nested case-control design with risk-set sampling was used to account for patients with HF who develop COPD during follow-up and for the varying nature of COPD severity over time. Using this approach, all cases were included in the analyses and were matched on their HF diagnosis date (±1 month) and duration of follow-up with up to 4 randomly sampled control participants from the HF cohort still at risk of the event. Control participants were eligible to be selected multiple times and later as a case, and exposure measurement was prior to the match date. This closely resembles the programming statements approach to Cox regression with time-varying covariates and produces unbiased estimates of hazard ratios.^16,17^ For the HF cohort (N = 50 114), cases were defined by all-cause mortality during follow-up; for the subcohort with hospital episode statistics–linked data (n = 30 061), cases were defined by a first hospitalization after but not including the HF diagnosis date. Date of death was based on clinical codes in CPRD using a CPRD algorithm.

### Measurement of COPD Severity

In patients with HF, COPD was identified by a clinical code and at least 1 COPD-related medication as recommended by the Global Initiative for Chronic Obstructive Lung Disease (GOLD) guidelines^18^ prior to the match date (see eTable 2 in the Supplement for COPD codes). A standard coding classification for COPD based on previous validation^19^ and expert clinical review was used and nonspecified bronchitis codes were excluded.

#### Medication Intensity Levels

The GOLD guidelines introduce an algorithm for the initiation and escalation for medication prescribing in COPD using a stepped-up approach based on severity. This approach combines airflow limitation stage with history of symptoms and exacerbations to define 4 severity groups (A-D). While the definition of COPD severity for prescribing has changed in the latest GOLD guidelines,^18^ the algorithms for medication prescribing are consistent. We used the GOLD guidelines to devise ordered medication intensity levels (Table 1). Exclusive levels ranged from the least severe that included short-acting anticholinergics or β_2_-antagonists only, to mono (GOLD group A), dual (GOLD group B or C), or triple (GOLD group D) therapy using a combination of long-acting anticholinergics, β_2_-antagonists, and inhaled corticosteroids. The GOLD groups B and C were combined owing to their substantial overlap in the guidelines. We also added 2 most severe levels that included oral corticosteroids and oxygen therapy, which are most commonly used following acute exacerbations or in patients with most severe disease. Each medication intensity level was defined by at least 1 of the specified medications prescribed in the 4-month timeframe before the match date to identify all patients with such long-term medication use.

**Table 1.** Classifications of COPD Medication Severity

Medication Intensity Levels^a^	Description^b^	GOLD Severity Groups
No medications	No COPD medications	
Short-term inhalers only	Any short-acting anticholinergic or β_2_-agonist but no other COPD medications	
Monotherapy	One long-acting bronchodilator (anticholinergic or β_2_-agonist) and/or short-acting inhalers but no other COPD medications	A
Dual therapy	An inhaled corticosteroid and 1 long-acting bronchodilator (β_2_-agonist or anticholinergic) and/or short-acting inhalers but no other COPD medications or both long-acting bronchodilators (β_2_-agonist and anticholinergic) and/or short-acting inhalers but no other COPD medications	B and C
Triple therapy	An inhaled corticosteroid and both long-acting bronchodilators (β_2_-agonist and anticholinergic) and/or short-acting inhalers but no other COPD medications	D
Noninhaled steroids	Noninhaled steroids can include any other COPD medications but not oxygen therapy	
Oxygen	Oxygen therapy can include any other COPD medications	

Abbreviations: COPD, chronic obstructive pulmonary disease; GOLD, Global Initiative for Obstructive Lung Disease.

^a^ Medication intensity levels for this study were devised using the GOLD pharmacological treatment escalation guidelines.^19^ These guidelines combine airflow limitation stage with symptom and exacerbation history to patients from groups A through D (A: less symptoms, low risk; B: more symptoms, low risk; C: less symptoms, high risk; D: more symptoms, high risk) with corresponding prescription regimens. The first and last 2 intensity levels were added to the GOLD groupings by the study investigators to define least and most severe prescribing groups to cover the full range of prescribing for patients with COPD. Medications were defined by at least 1 prescription in the 4 months prior to the match date. Severity levels were mutually exclusive.

^b^ Methylxanthines may replace 1 of the long-acting bronchodilators.

#### COPD Airflow Limitation Stages

The GOLD guidelines recommend the use of forced expiratory volume in 1 second (FEV_1_) to measure the severity of airflow limitation in COPD.^18^ Patients with COPD with routinely recorded spirometry were stratified by 4 severity stages recommended by GOLD: mild, FEV_1_ 80% or more predicted; moderate, 50% or more FEV_1_ but less than 80% predicted; severe, 30% or more FEV_1 _but less than 50% predicted; and very severe, FEV_1_ less than 30% predicted. The most recent FEV_1_ prior to the match date was used within a maximum 3-year timeframe. The median FEV_1_ measurement time was 295 days (range, 137-524 days) prior to mortality matching and 247 days (range, 103-280 days) prior to admission matching.

### Measurement of Confounders

For potential confounders^20^ (eTable 3 in the Supplement), the most recent measurement before the match date was used. Medication measurements were defined by at least 1 prescription in a 4-month timeframe prior to the match date.

### Statistical Analysis

Statistical analyses were performed for the 2 outcomes separately. First, patient and clinical characteristics were compared between cases and matched controls. To build the multivariable models, quadratic terms were added for nonlinear continuous variables and first-order interactions were tested between COPD and main confounders. Multiple imputation was performed for the missing confounder data (<15%; eTable 4 in the Supplement) using matching variables and full-conditional specification in StataMP, version 14 (StataCorp) to produce 10 imputed data sets, which were combined using Rubin rule.^21^

Next, unadjusted and adjusted conditional logistic regressions were used to compare patients with HF and COPD vs patients with HF but without COPD for the outcome event. The COPD group was stratified into subgroups by measures of recent medication treatment intensity and measures of airflow limitation. The subgroups with COPD were compared with the reference group, patients with HF but without COPD. All estimates were presented as adjusted odds ratios (AORs) with 95% CIs. Significant difference among groups with COPD by medication treatment intensity or airflow limitation was indicated where confidence intervals for estimates did not overlap.^22^

Sensitivity analyses were performed to investigate COPD association with outcomes stratified by those with and without a spirometry recording and those with an HF index date prior to or after April 1, 2006. This date was 2 years prior to the introduction of incentives to improve use of echocardiogram and spirometry diagnostics in the United Kingdom’s Quality and Outcomes Framework.

## Results

### Study Population

There were 50 114 patients with incident HF, the median age was 79 years (interquartile range, 71-85 years), 46% were women, and 18 478 patients (13.8%) had COPD. Deaths occurred in 26 729 patients (53%) over a median follow-up of 2.6 years (interquartile range, 0.8-5.0 years) and all 26 729 of the patients who died were matched with up to 4 controls still alive (n = 106 916). Of the linked hospitalization sample (n = 30 061), 24 339 patients (81%) had a first hospitalization during a median follow-up of 82 days (interquartile range, 12-435 days) and all 24 339 of these were matched with up to 4 control patients who had not yet been admitted (n = 86 450). Cases for both outcomes were older, with more comorbidities but fewer cardiovascular medications and a lower body mass index, cholesterol level, hemoglobin level, and blood pressure than controls (Table 2). For mortality, 4630 cases (17.3%) had COPD compared with 13 848 controls (13.0%) and for first hospitalization, 3230 cases (13.3%) had COPD vs 8673 controls (10.0%). β-Blockers were prescribed for 74 221 (55.5%) of the total sample (Table 2) and this was lower in the groups with COPD and more severe airflow limitation (16%-30%) (eTable 5 in the Supplement). Most patients with HF and COPD were prescribed inhaler medications (10 510 [56.9%]) but 2359 (13%) from the mortality sample and 2093 (18%) from the hospitalization sample had not been prescribed any COPD-related medications in the 4 months prior to their match dates (Table 3). A quarter of patients with HF and COPD were prescribed oral steroids with no oxygen (mortality: 2817 [23.7%]; hospitalization: 2673 [22.5%]) and 3% were prescribed oxygen therapy (mortality: 343 [2.9%]; hospitalization: 337 [2.8%]), and both were more likely in the cases than controls for both outcomes. A spirometry recording was available only for 8515 patients (46.1%) with COPD in a 3-year timeframe prior to the mortality match date and 4652 patients (39.1%) prior to the hospitalization match date. Of the patients with HF and COPD, those with a spirometry measure were more likely to be older, male, more deprived, lower body mass index, cholesterol, and hemoglobin than those without (eTable 6 in the Supplement). Those patients were also most likely to have moderate to severe airflow limitation (70%) and less likely to have mild airflow limitation (15%). Very severe airflow limitation was present in 10% of patients (Table 3).

**Table 2.** Patient Characteristics by Outcomes

Patient Characteristics	No. (%)					
	Mortality Sample			Hospitalization Subsample		
	All (n = 133 645)	Cases (n = 26 729)	Controls (n = 106 916)	All (n = 110 789)	Cases (n = 24 339)	Controls (n = 86 450)
Age, median (IQR), y	79 (71-85)	83 (76-88)	78 (70-84)	78 (70-84)	79 (72-85)	78 (70-84)
Women	61 732 (46.2)	12 974 (48.5)	48 758 (45.6)	53 804 (48.6)	11 388 (46.8)	42 416 (49.1)
IMD quintile						
1	15 908 (20.0)	3064 (18.7)	12 884 (20.4)	22 559 (20.4)	4668 (19.2)	17 891 (20.7)
2	18 089 (22.8)	3708 (22.6)	14 381 (22.8)	26 609 (24.0)	5614 (23.1)	20 995 (24.3)
3	16 666 (21.0	3570 (21.8)	13 096 (20.8)	23 009 (20.8)	5153 (21.2)	17 856 (20.6)
4	16 451 (20.7)	3427 (20.9)	13 024 (20.7)	22 575 (20.4)	5068 (20.8)	17 507 (20.3)
5	12 269 (15.5)	2613 (16.0)	9656 (15.3)	16 037 (14.5)	3836 (15.8)	12 201 (14.1)
BMI, median (IQR)	26.9 (23.5-31.0)	25.4 (22.1-29.3)	27.3 (24.0-31.3)	27.2 (23.9-31.2)	26.6 (23.4-30.4)	27.2 (24.1-31.2)
Cholesterol, mean (SD), mg/dL	175.2 (45.7)	171.9 (47.3)	176.0 (45.2)	181.7 (46.4)	183.0 (44.6)	180.0 (45.9)
Hb, mean (SD), g/dL	13.0 (1.9)	12.3 (2.0)	13.1 (1.8)	13.3 (1.7)	12.9 (1.9)	13.4 (1.6)
Systolic BP, mean (SD), mm Hg	131.4 (20.5)	126.9 (22.3)	132.5 (19.9)	135.6 (20.2)	134.1 (21.5)	136.0 (19.8)
Diastolic BP, mean (SD), mm Hg	73.3 (11.4)	71.1 (12.0)	73.9 (11.1)	75.7 (11.2)	74.7 (12.0)	75.9 (11.0)
Diuretics	103 283 (77.3)	21 574 (80.7)	81 709 (76.4)	79 860 (72.1)	17 023 (69.9)	62 837 (72.7)
β-Blocker	74 221 (55.5)	12 171 (45.5)	62 050 (58.0)	44 467 (40.1)	8893 (36.5)	35 574 (41.2)
ACEi	74 373 (55.7)	12 207 (45.7)	62 166 (58.1)	63 907 (57.7)	12 477 (51.3)	51 430 (59.5)
ARB	22 753 (17.0)	3170 (11.9)	19 583 (18.3)	19 324 (17.4)	3722 (15.3)	15 602 (18.1)
ACEi or ARB	94 547 (70.7)	15 079 (56.4)	79 468 (74.3)	80 420 (72.6)	15 645 (64.3)	64 755 (74.9)
Aspirin	99 180 (74.2)	20 151 (75.4)	79 029 (73.9)	74 639 (67.4)	16 657 (68.4)	57 982 (67.1)
AA	23 863 (17.9)	5610 (21.0)	18 253 (17.1)	13 272 (12.0)	3230 (13.3)	10 042 (11.6)
COPD	18 478 (13.8)	4630 (17.3)	13 848 (13.0)	11 903 (10.7)	3230 (13.3)	8673 (10.0)
Diabetes	31 962 (23.9)	6714 (25.1)	25 248 (23.6)	21 291 (19.2)	5577 (22.9)	15 714 (18.2)
Renal disease, eGFR <60 mm/min/m^2^	66 301 (55.4)	15 827 (66.2)	50 474 (52.7)	49 768 (51.3)	12 053 (56.5)	37 714 (49.8)
Atrial fibrillation	48 968 (36.6)	10 108 (37.8)	38 860 (36.4)	35 298 (31.9)	8365 (34.4)	26 933 (31.2)
Hypertension	77 453 (58.0)	15 403 (57.6)	62 055 (58.0)	62 502 (56.4)	13 895 (57.1)	48 607 (56.2)
Ischemic heart disease	58 711 (49.1)	11 150 (41.7)	43 159 (40.4)	37 782 (34.1)	9283 (38.1)	28 499 (33.0)
Myocardial infarction	35 998 (26.9)	7509 (28.1)	28 489 (26.7)	23 670 (21.4)	6171 (25.4)	17 499 (20.2)
Smoking status						
Current	14 941 (11.2)	3125 (11.7)	11 816 (11.1)	12 184 (11.0)	3057 (12.6)	9127 (10.6)
Not current	61 874 (46.3)	12 541 (46.9)	49 333 (46.1)	53 150 (48.0)	11 236 (46.2)	41 914 (48.5)
Past smoker	56 830 (42.5)	11 063 (41.4)	45 767 (42.8)	45 455 (41.0)	10 046 (41.3)	35 409 (41.0)
Alcohol status						
Current	92 456 (69.2)	17 661 (66.1)	74 795 (70.0)	81 695 (73.7)	17 489 (71.9)	64 206 (74.3)
Not current	34 648 (25.9)	7651 (28.6)	26 997 (25.3)	24 741 (22.3)	5801 (23.8)	18 940 (21.9)
Past drinker	6541 (4.9)	1417 (5.3)	5124 (4.8)	4353 (3.9)	1049 (4.3)	3304 (3.8)

Abbreviations: AA, aldosterone antagonist (spironolactone or eplerenone); ACEi, angiotensin-converting enzyme inhibitor; ARB, angiotensin-receptor blocker; BMI, body mass index (calculated as weight in kilograms divided by height in meters squared); BP, blood pressure; COPD, chronic obstructive pulmonary disease; eGFR, estimated glomerular filtration rate; Hb, hemoglobin; IMD, index multiple deprivation (1, least deprived and 5, most deprived); IQR, interquartile range.

SI conversion factor: To convert cholesterol to millimoles per liter, multiply by 0.0259; Hb to grams per liter, multiply by 10.

**Table 3.** COPD Severity Measures in Those With HF and COPD

COPD Severity	Mortality Sample			Hospitalization Subsample		
	HF With COPD (n = 18 478)	COPD Cases (n = 4630)	COPD Controls (n = 13 848)	HF With COPD (n = 11 903)	COPD Cases (n = 3230)	COPD Controls (n = 8673)
Medication intensity levels, No. (%)^a^						
No medications	2359 (12.8)	561 (12.2)	1798 (13.0)	2093 (17.6)	498 (15.4)	1595 (18.4)
Short-term inhalers only	1624 (8.8)	380 (8.2)	1244 (9.0)	1192 (10.0)	301 (9.3)	891 (10.3)
Monotherapy	2417 (13.1)	542 (11.7)	1875 (13.5)	1725 (14.5)	446 (13.8)	1279 (14.8)
Dual therapy	3329 (18.0)	664 (14.3)	2665 (19.2)	2305 (19.4)	539 (16.7)	1766 (20.4)
Triple therapy	3140 (17.0)	699 (15.0)	2441 (17.6)	1588 (13.3)	393 (12.2)	1195 (13.8)
Any inhalers only	10 510 (56.9)	2285 (49.4)	8225 (59.4)	6801 (57.2)	1679 (52.0)	5131 (59.2)
Oral corticosteroids but no oxygen	2817 (23.7)	1415 (30.6)	3329 (24.0)	2673 (22.5)	895 (27.7)	1778 (20.5)
Oxygen therapy	343 (2.9)	369 (8.0)	496 (3.6)	337 (2.8)	158 (4.9)	169 (2.0)
Spirometry	8515 (53.9% missing)	2859	5656	4652 (60.9% missing)	1453	3199
FEV_1_, median (IQR), % predicted^b^	54.1 (39.7-70.0)	51.9 (37.7-68.5)	55.2 (40.6-70.6)	54.1 (39.7-70.0)	53.0 (38.5-70.6)	53.0 (38.4-69.0)
COPD GOLD airflow limitation stage^c^						
Stage 1: FEV_1 _≥80% normal (mild)	1284 (15.1)	396 (13.9)	888 (15.7)	721 (15.5)	237 (16.3)	484 (15.1)
Stage 2: FEV_1_ 50%-79% normal (moderate)	3721 (43.7)	1140 (39.9)	2581 (45.6)	1896 (40.8)	585 (40.3)	1311 (41.0)
Stage 3: FEV_1_ 30%-49% normal (severe)	2679 (31.5)	989 (34.6)	1690 (29.9)	1512 (32.5)	464 (31.9)	1048 (32.8)
Stage 4: FEV_1_ <30% normal (very severe)	831 (9.8)	334 (11.7)	497 (8.8)	523 (11.2)	167 (11.5)	356 (11.2)

Abbreviations: COPD, chronic obstructive pulmonary disease; FEV_1_, forced expiratory volume in 1 second; IQR, interquartile range; GOLD, Global Initiative for Obstructive Lung Disease; HF, heart failure.

^a^ Monotherapy, dual therapy, or triple therapy; respectively 1, 2, or 3 of the following: long-acting β_2_-antagonist, long-acting cholinergic, methylxanthines, inhaled corticosteroid either individually or in combination inhalers.

^b^ Most recently recorded FEV_1_ (% predicted) before the match date was used to measure airflow limitation within a maximum of a 3-year timeframe.

^c^ The COPD GOLD severity stages according to airflow limitation.

### COPD in HF and Outcomes

Presence of HF and COPD were significantly associated with all-cause mortality (AOR, 1.31; 95% CI, 1.26-1.36) and with first hospitalization (AOR, 1.33; 95% CI, 1.26-1.39) (Table 4). These associations were not affected by β-blockers (*P* value for interaction >.05) but mortality risk was significantly higher in women (AOR, 1.41; 95% CI, 1.30-1.53) than men (AOR, 1.26; 95% CI, 1.18-1.34) (*P* value for interaction = .01) (eTable 7 in the Supplement). When the group with COPD was stratified by the presence or absence of spirometry data and compared with patients with HF without COPD (reference group), patients without spirometry data had decreased mortality (AOR, 0.87; 95% CI, 0.82-0.92) and reduced risk of admission (AOR, 1.20; 95% CI, 1.12-1.26). Within the group with COPD, patients with spirometry recording had significantly increased mortality (AOR, 2.22; 95% CI, 2.06-2.39) and hospitalization (AOR, 1.28; 95% CI, 1.17-1.40) compared with those without spirometry recording (eTable 8 in the Supplement).

**Table 4.** Chronic Obstructive Pulmonary Disease Associations With All-Cause Mortality and Hospitalization in Patients With HF, Stratified by Drug and Spirometry Measures

	Mortality Sample		Hospitalization Subsample	
	Unadjusted OR (95% CI)	Adjusted OR (95% CI)^a^	Unadjusted OR (95% CI)	Adjusted OR (95% CI)^a^
HF with no COPD	1 [Reference]	1 [Reference]	1 [Reference]	1 [Reference]
COPD	1.41 (1.36-1.46)	1.31 (1.26-1.36)	1.41 (1.36-1.46)	1.33 (1.26-1.39)
COPD group stratified by medication intensity levels^b^				
HF with no COPD	1 [Reference]	1 [Reference]	1 [Reference]	1 [Reference]
COPD with no medications	1.31 (1.19-1.44)	1.23 (1.11-1.37)	1.17 (1.06-1.30)	1.16 (0.04-1.30)
COPD with short-term inhalers only	1.28 (1.14-1.44)	1.10 (1.11-1.25)	1.25 (1.10-1.43)	1.28 (1.10-1.47)
COPD with monotherapy	1.22 (1.11-1.34)	1.09 (0.98-1. 21)	1.28 (1.14-1.43)	1.26 (1.12-1. 42)
COPD with dual therapy	1.05 (0.96-1.14)	0.99 (0.90-1.09)	1.13 (1.02-1.24)	1.09 (0.98-1.21)
COPD with triple therapy	1.21 (1.11-1.31)	1.17 (1.06-1.29)	1.21 (1.07-1.35)	1.17 (1.03-1.33)
COPD with noninhaled steroids but no oxygen therapy	1.79 (1.68-1.91)	1.69 (1.57-1.81)	1.79 (1.65-1.94)	1.75 (1.59-1.92)
COPD with oxygen therapy	3.14 (2.74-3.60)	2.82 (2.42-3.28)	3.40 (2.73-4.23)	2.84 (1.22-3.63)
COPD with any inhalers only	1.17 (1.11-1.23)	1.08 (1.02-1.14)	1.17 (1.11-1.23)	1.20 (1.12-1.28)
COPD group stratified by airflow limitation (GOLD stages)^c^				
HF with no COPD	1 [Reference]	1 [Reference]	1 [Reference]	1 [Reference]
COPD GOLD stage				
Stage 1, FEV_1_ ≥80% normal	1.88 (1.66-2.12)	1.63 (1.42-1.87)	1.67 (1.43-1.95)	1.59 (1.33-1.90)
Stage 2, FEV_1_ 50%-79% normal	1.87 (1.74-2.01)	1.69 (1.56-1.83)	1.57 (1.43-1.74)	1.50 (1.34-1.67)
Stage 3, FEV_1_ 30%-49% normal	2.47 (2.27-2.68)	2.21 (2.01-2.42)	1.58 (1.41-1.77)	1.48 (1.31-1.68)
Stage 4, FEV_1_ <30% normal	2.84 (2.47-3.27)	2.93 (2.49-3.43)	1.64 (1.366-1.97)	1.73 (1.40-2.12)

Abbreviations: COPD, chronic obstructive pulmonary disease; FEV_1_, forced expiratory volume in 1 second; GOLD; Global Initiative for Obstructive Lung Disease; HF, heart failure; OR, odds ratio.

^a^ Adjusted models included age, sex, body mass index, cholesterol level, estimated glomerular filtration rate,^2^ hemoglobin level,^2^ systolic blood pressure,^2^ diuretics, β-blocker, angiotensin-converting enzyme inhibitor, angiotensin-receptor blocker, aldosterone antagonist (spironolactone or eplerenone), aspirin, atrial fibrillation, hypertension, ischemic heart disease, myocardial infarction, diabetes, smoking, alcohol use, and previous hospitalization in 12 months (hospitalization models only).

^b^ Drug measures were defined by at least 1 prescription in a 4-month timeframe prior to the match date. Intensity levels are mutually exclusive. Monotherapy, dual therapy, or triple therapy are, respectively, 1, 2, or 3 of a long-acting β_2_-antagonist, long-acting cholinergic, methylxanthines, or inhaled corticosteroid (individually or in combination inhalers) but no noninhaled steroids or oxygen therapy.

^c^ The most recently recorded FEV_1_ prior to the match date was used within a maximum of a 3-year timeframe.

### COPD Medication Intensity in HF and Mortality

The adjusted associations between COPD and mortality differed significantly by levels of COPD medication intensity. Patients with no COPD medications (AOR, 1.23; 95% CI, 1.11-1.37) in the 4 months before outcome or receiving oral corticosteroids (AOR, 1.69; 95% CI, 1.57-1.81) or oxygen therapy (AOR, 2.82; 95% CI, 2.42-3.28) had the strongest risk associations with mortality. No significant association with mortality was found for those receiving monotherapy or dual therapy (GOLD groups A-C); however, a significant risk increase was observed for those receiving triple therapy (GOLD group D) (AOR, 1.17; 95% CI, 1.06-1.29) (Table 4).

### COPD Medication Intensity in HF and Hospitalization

The adjusted associations between COPD and hospitalization also significantly increased from those receiving triple therapy (AOR, 1.17; 95% CI, 1.03-1.33) to oral corticosteroids (AOR, 1.75; 95% CI, 1.59-1.92), to oxygen therapy (AOR, 2.84; 95% CI, 1.22-3.63). Compared with patients with HF but without COPD, there was no significant association with hospitalization for patients with COPD who were not prescribed COPD medications in the 4 months prior to admission or who were prescribed dual therapy, whereas those prescribed short-term inhalers only or monotherapy had increased risk (AOR, 1.28; 95% CI, 1.10-1.47; AOR, 1.26; 95% CI, 1.12-1.42, respectively).

### COPD Stratified by Airflow Limitation in HF and Mortality

When patients with HF and COPD were stratified by GOLD stages according to airflow limitation measured by FEV_1_ and compared with the HF reference group without COPD, there was a significant difference in estimates among the severity stages. Patients with severe (30% ≤ FEV_1_ < 50%) and very severe (FEV_1_ < 30%) COPD had the highest risk association with mortality (AOR, 2.21; 95% CI, 2.01-2.42; AOR, 2.93; 95% CI, 2.49-3.43, respectively). Patients with mild and moderate COPD (GOLD stages 1 and 2) were also associated with an increased risk of mortality (AOR, 1.63; 95% CI, 1.42-1.87; AOR, 1.69; 95% CI, 1.56-1.83, respectively).

### COPD Stratified by FEV_1_ in HF and Hospitalization

All groups with COPD stratified by airflow limitation were significantly associated with increased risk of hospitalization (Table 4). The most severe group (GOLD stage 4) had the highest risk (AOR, 1.73; 95% CI, 1.40-2.12), but there was no significant difference in risk among groups.

## Discussion

This study, to our knowledge, was the largest population-based study to investigate the association between COPD and outcomes in an incident cohort of more than 50 000 patients with HF. Uniquely, in the HF community setting, we have shown that the increased risk of death and hospitalization associated with COPD significantly differs by medication intensity and the severity of airflow limitation. Chronic obstructive pulmonary disease was not associated with any increased risk of death when patients were managed by inhaler therapies, until prescribing intensity reached triple inhaler therapy and risks of both outcomes were significantly higher in those prescribed oral corticosteroids and oxygen therapy. Spirometry assessment may be underused in the community but indicated a more severe HF and COPD group, with worse outcomes for those with the most severe airflow limitation. These findings provide key evidence for risk stratifying patients with HF and COPD in the community, where most patients are routinely managed.

Our findings have 4 key clinical implications. First, to improve HF prognosis, the importance of accurately identifying and effectively managing comorbidities is increasingly recognized.^23^ Our findings show that 1 in 7 patients with HF in the community also has COPD, which carries a 30% increase in risk of death and hospitalization compared with patients with HF but without COPD. Prevalence of COPD in the community was similar to other clinical database studies^5,8^ but was much lower than in HF studies using formal spirometry screening, which indicates a potential prevalence of up to 40%.^6,7,10^ This means the number of patients with HF and COPD in the community is likely to be much higher than was recorded,^24,25^ with a potentially even greater effect on outcomes. The prescribing of β-blockers was particularly low for patients with COPD but was comparable with other studies.^7,8^ However, adjustment did not explain the association between COPD and risk of mortality or hospitalization.

Spirometry data were not recorded for up to a third of patients with COPD in the community^26^ and were missing for half of patients with HF and COPD during the study period. The study found that, in those without a spirometry recording, the COPD association with mortality was protective and with hospitalization was reduced. Given that the common symptom of breathlessness potentially drives spirometry assessment, it is likely that the COPD group without spirometry includes those with milder-severity COPD as well as less severe HF compared with the reference group. An alternative consideration is the potential misclassification of COPD in this group in the absence of pulmonary function assessment, particularly at lower grades of severity. The diagnosis of COPD in patients with HF is complicated by nonspecific shared symptoms such as breathlessness and spirometry is required for accurate diagnosis,^27^ which can be particularly challenging in the community setting. While we used specific clinical COPD diagnostic codes that have demonstrated high precision in CPRD,^19^ this study highlights an urgent need to improve routine assessment of lung function for all patients with HF and COPD in the community.

Second, our findings show that COPD-associated risk differed significantly according to medication intensity in patients with HF and so provided a potential indicator of disease progression. Patients with HF and COPD who were receiving inhaler therapies (GOLD groups A-C) had comparable mortality risk with patients with HF but without COPD. Similarly, hospitalization risk was not increased for those prescribed dual therapy (GOLD groups B and C), but was increased for patients with COPD prescribed short-term inhalers only or monotherapy, which may reflect newer-onset, unstable COPD in these groups. While the inhaler groups were overall at lower risk than those receiving oral corticosteroids and oxygen, there was poor differentiation in risk among inhaler groups for both outcomes. This finding is corroborated by previous evidence showing poor discrimination of GOLD severity classifications for mortality.^28^ Newer GOLD guidelines focus on symptoms-based severity assessment, which creates new challenges in patients with HF who share breathlessness and functional limitation as predominant symptoms. Consequently, this may lead to overtreatment with pulmonary inhaler therapies in patients with HF and COPD.

A third of patients with COPD were prescribed oral steroids or oxygen therapy and had up to a 3-fold increase in risk of death or hospitalization. In COPD, corticosteroids have been associated with increased mortality risk, which may relate to their adverse effect on other comorbidities such as diabetes,^29^ weakened respiratory muscle strength after prolonged therapy, or retention of sodium and water,^30^ all of which might lead to exacerbations of HF. Alternatively, short-term prescribing of oral corticosteroids or oxygen therapy are usually a result of acute COPD exacerbations, which are also potentially associated with mortality^31^ and are a likely pseudomarker of more severe COPD disease. Of interest was the higher risk of mortality for patients with COPD prescribed no related medications in the 4 months prior to death, which might indicate end-stage HF severity when de-prescribing may occur.

Third, comparing patients with HF and COPD with those without COPD, the risk of mortality increased with more severe airflow limitation from GOLD stages 1 through 4. Other hospital studies have found no association between COPD in HF with mortality for mild^6,7^ or moderate^10^ airflow limitation or for overall COPD.^6,10^ These studies used formal spirometry screening in patients with HF to identify and assess COPD severity and included a much higher proportion of patients with mild-severity COPD. In our community cohort of patients with COPD and spirometry data, just under half were in the most severe 2 stages, which might partly explain these differences, combined with the higher power of a large sample. The lack of risk stratification using FEV_1_ for the hospitalization outcome is perhaps not surprising given that most first admissions may have been driven by the new HF diagnosis, but further research in a prevalent HF cohort would determine the prognostic value of FEV_1_ for admissions.

Of note was the higher risk of both outcomes in the mildest airflow limitation group than in the overall group vs COPD. This indicates that use of spirometry assessment in the community setting was itself associated with a higher-risk group. On further examination, the patients with COPD with spirometry data had a worse risk profile than those without, meaning that risk factors as well as COPD severity may drive some of the clinical decisions to request spirometry. While the COPD associations were adjusted for these risk factors, the worse risk profile in the spirometry group is also likely to indicate worse HF severity.

Fourth, COPD in women with HF was associated with a 15% higher risk of death than in men. Other studies have previously reported higher mortality rates in women than men, despite comparable lung function and even lower levels of smoking.^32^ Similarly, while mortality rates are decreasing for men with COPD, they are increasing in women.^33^ Reasons for higher mortality in women range from genetics and physiology, to delayed diagnosis, to suboptimal treatment responses^34^ that are accelerated increasing in postmenopausal age.^35^ Our findings add new evidence that sex differences also occur in patients with HF and indicate an important group for treatment optimization.

### Limitations

Our study, to our knowledge, is the largest to date to investigate COPD in the general HF population. The UK national database of routinely recorded medication and clinical data meant that we could devise and test guideline-driven COPD severity groups using readily accessible information and adjust for a wide range of patient, medication, and clinical factors. While the CPRD is a clinically validated and globally used epidemiological database,^14^ routinely collected data can be subject to measurement error. We based the HF cohort on validated clinical codes that have high precision,^15^ but these data did not include ejection fraction to determine the HF phenotype and may still include some misclassification of HF. Further investigation in a HF cohort with brain natriuretic peptide or echocardiographic data would be important.^36^ Validated COPD clinical codes and related medications improved the precision of diagnosis,^19^ but this does not negate undiagnosed COPD in the community setting. However, any such misclassification owing to underascertainment of COPD is likely to bias the associations toward the null value.

The FEV_1_ can be influenced by HF status^37^ that can mimic obstruction^24^ resulting in approximately a quarter of patients with HF but without COPD having reduced FEV_1_,^10,38^ leading to measurement error of COPD severity. Furthermore, where there is an imbalance in the reduction of FEV_1_ and forced vital capacity as a consequence of pulmonary congestion, a spurious obstructive pattern can occur, resulting in misdiagnosis of COPD. It is therefore particularly important in HF to consider hyperventilation using body plethysmography as an alternative approach to COPD diagnosis and assessment.^4^ Lack of ejection fraction data meant that we could not investigate the study findings in different HF phenotypes or take account of HF severity in the COPD associations. So, while we could show the direction and pattern of COPD associations stratified by measures of COPD severity in a real-world community setting, we were not able to fully disentangle COPD severity from HF severity. Further validation of the study findings in a prospective community HF cohort with pulmonary and cardiac function testing is required.

## Conclusions

This study has shown that routine measures of COPD severity in the community population with HF offer potentially important prognostic tools for risk stratification. Optimal treatment for HF and COPD becomes challenging when they occur together as evident by the study’s findings. The results generate the hypothesis that some of the adverse outcomes in the HF population with COPD could be improved by targeting better diagnosis of both, optimizing drug treatment for both, and identifying patients with the greatest severity of HF and COPD for early aggressive treatment or close monitoring.

## Notes

Supplement. **eFigure.** Flowchart of the Patient Selection Process **eTable 1.** Heart Failure Selection Code Set **eTable 2.** Chronic Obstructive Pulmonary Disease Code Set **eTable 3.** Potential Confounders **eTable 4.** Missing Data Preimputation **eTable 5.** Characteristics of Patients With HF and COPD by Airflow Limitation in Those With Spirometry Data **eTable 6.** Characteristics of Patients With HF and COPD by Spirometry Availability **eTable 7.** Interactions **eTable 8.** Sensitivity Analyses
