# Supplementary material for: Association of Medication Intensity and Stages of Airflow Limitation With the Risk of Hospitalization or Death in Patients With Heart Failure and Chronic Obstructive Pulmonary Disease
Source: JAMA Netw Open. 2018 Dec 14;1(8):e185489. doi: 10.1001/jamanetworkopen.2018.5489 (PMC6324325; doi:10.1001/jamanetworkopen.2018.5489)
Supplement: Supplement. — eFigure. Flowchart of the Patient Selection Process eTable 1. Heart Failure Selection Code Set eTable 2. Chronic Obstructive Pulmonary Disease Code Set eTable 3. Potential Confounders eTable 4. Missing Data Preimputation eTable 5. Characteristics of Patients With HF and COPD by Airflow Limitation in Those With Spirometry Data eTable 6. Characteristics of Patients With HF and COPD by Spirometry Availability eTable 7. Interactions eTable 8. Sensitivity Analyses [file jamanetwopen-1-e185489-s001.pdf]

Supplementary Online Content

Lawson CA, Mamas MA, Jones PW, et al. Association of medication intensity and stages of airflow limitation with the risk of hospitalization or death in patients with heart failure and chronic obstructive pulmonary disease. *JAMA Netw Open*. 2018;1(8):e185489. doi:10.1001/jamanetworkopen.2018.5489

eFigure. Flowchart of the Patient Selection Process

eTable 1. Heart Failure Selection Code Set

eTable 2. Chronic Obstructive Pulmonary Disease Code Set

eTable 3. Potential Confounders

eTable 4. Missing Data Preimputation

eTable 5. Characteristics of Patients With HF and COPD by Airflow Limitation in Those With Spirometry Data

eTable 6. Characteristics of Patients With HF and COPD by Spirometry Availability

eTable 7. Interactions

eTable 8. Sensitivity Analyses

This supplementary material has been provided by the authors to give readers additional information about their work.

© 2018 Lawson CA et al. *JAMA Network Open*.

eFigure. Flowchart of the Patient Selection Process

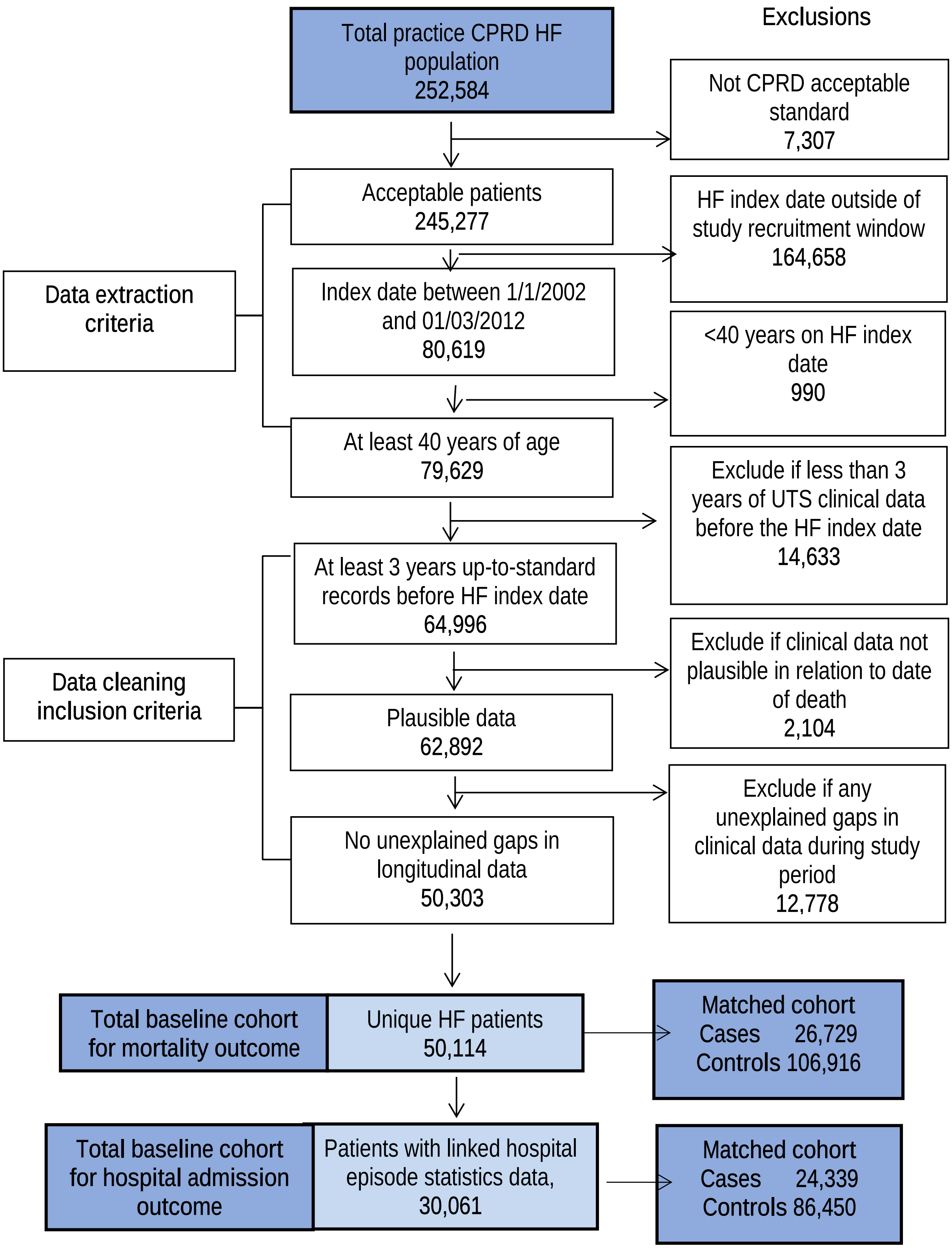

© 2018 Lawson CA et al. *JAMA Network Open*.

eTable 1. Heart Failure Selection Code Set

CPRD Medcode	Read code	Read term
398	G580.00	Congestive heart failure
884	G581.00	Left ventricular failure
2062	G58..00	Heart failure
2906	G580.11	Congestive cardiac failure
4024	G58z.00	Heart failure NOS
1223	G58..11	Cardiac failure
5942	G581.13	Impaired left ventricular function
5255	G581000	Acute left ventricular failure
32671	G580100	Chronic congestive heart failure
10079	G580.12	Right heart failure
9524	G580.14	Biventricular failure
17278	G58z.12	Cardiac failure NOS
23707	G580000	Acute congestive heart failure
10154	G580.13	Right ventricular failure
27964	G582.00	Acute heart failure
27884	G580200	Decompensated cardiac failure
23481	G581.11	Asthma – cardiac
43618	G581.12	Pulmonary oedema – acute
11424	G580300	Compensated cardiac failure
22262	G58z.11	Weak heart

© 2018 Lawson CA et al. *JAMA Network Open*.

12590	G583.00	Heart failure with normal ejection fraction
101138	G580400	Congestive heart failure due to valvular disease
94870	G584.00	Right ventricular failure
104275	G583.11	HFNEF - heart failure with normal ejection fraction
101137	G1yz100	Rheumatic left ventricular failure
9913	101..00	Heart Failure confirmed
21837	G232.00	Hypertensive heart&renal dis wth (congestive) heart failure

© 2018 Lawson CA et al. *JAMA Network Open*.

eTable 2. Chronic Obstructive Pulmonary Disease Code Set

CPRD Medcode	**Read code**	**Read Term**
**1001**	H3...00	Chronic obstructive pulmonary disease
**998**	H3...11	Chronic obstructive airways disease
**10863**	H36..00	Mild chronic obstructive pulmonary disease
**5710**	H3z..00	Chronic obstructive airways disease NOS
**10802**	H37..00	Moderate chronic obstructive pulmonary disease
**794**	H32..00	Emphysema
**9876**	H38..00	Severe chronic obstructive pulmonary disease
**93568**	H39..00	Very severe chronic obstructive pulmonary disease
**14798**	H312100	Emphysematous bronchitis
**12166**	H3y..00	Other specified chronic obstructive airways disease
**33450**	H32z.00	Emphysema NOS
**37247**	H3z..11	Chronic obstructive pulmonary disease NOS
**26306**	H320.00	Chronic bullous emphysema
**10980**	H322.00	Centrilobular emphysema
**23492**	H320z00	Chronic bullous emphysema NOS
**44525**	H312z00	Obstructive chronic bronchitis NOS
**11287**	66YM.00	Chronic obstructive pulmonary disease annual review
**9520**	66YB.00	Chronic obstructive pulmonary disease monitoring
**28755**	9Oi0.00	Chronic obstructive pulmonary disease monitoring 1st letter
**18621**	66YL.00	Chronic obstructive pulmonary disease follow-up
**34202**	9Oi1.00	Chronic obstructive pulmonary disease monitoring 2nd letter
**18476**	66YL.11	COPD follow-up
**34215**	9Oi2.00	Chronic obstructive pulmonary disease monitoring 3rd letter
**18792**	9Oi..00	Chronic obstructive pulmonary disease monitoring admin
**37371**	66YD.00	Chronic obstructive pulmonary disease monitoring due
**26018**	66YS.00	Chronic obstructive pulmonary disease monitoring by nurse
**45771**	66Yh.00	Chronic obstructive pulmonary disease does not disturb sleep
**45998**	66YT.00	Chronic obstructive pulmonary disease monitoring by doctor
**3243**	H31..00	Chronic bronchitis
**15157**	H31z.00	Chronic bronchitis NOS
**15626**	H310000	Chronic catarrhal bronchitis
**5798**	H312000	Chronic asthmatic bronchitis
**27819**	H312.00	Obstructive chronic bronchitis
**25603**	H310.00	Simple chronic bronchitis
**5909**	H312011	Chronic wheezy bronchitis
**11150**	H311.00	Mucopurulent chronic bronchitis
**26125**	H312300	Bronchiolitis obliterans

© 2018 Lawson CA et al. *JAMA Network Open*.

**45089**	H31y100	Chronic tracheobronchitis
**16410**	H32yz00	Other emphysema NOS
**40788**	H32y.00	Other emphysema
**40159**	H311000	Purulent chronic bronchitis
**61118**	H310z00	Simple chronic bronchitis NOS
**104608**	H3A..00	End stage chronic obstructive airways disease
**24248**	H313.00	Mixed simple and mucopurulent chronic bronchitis
**61513**	H311z00	Mucopurulent chronic bronchitis NOS
**63479**	H32y200	MacLeod's unilateral emphysema
**66043**	H31y.00	Other chronic bronchitis
**68066**	H31yz00	Other chronic bronchitis NOS
**46578**	H321.00	Panlobular emphysema
**60188**	H320200	Giant bullous emphysema
**56860**	H320000	Segmental bullous emphysema
**68662**	H320100	Zonal bullous emphysema
**99536**	H320300	Bullous emphysema with collapse
**67040**	H3y..11	Other specified chronic obstructive pulmonary disease
**37959**	H311100	Fetid chronic bronchitis
**70787**	H32y100	Atrophic (senile) emphysema
**59263**	H32y111	Acute interstitial emphysema
**103733**	H320311	Tension pneumatocoele
**92955**	H32y000	Acute vesicular emphysema
**1446**	H312200	Acute exacerbation of chronic obstructive airways disease
**28743**	66Yf.00	Number of COPD exacerbations in past year
**18501**	66YI.00	COPD self-management plan given
**7884**	H3y1.00	Chron obstruct pulmonary dis wth acute exacerbation, unspec
**11019**	8H2R.00	Admit COPD emergency
**42313**	679V.00	Health education - chronic obstructive pulmonary disease
**101042**	8BMW.00	Issue of chronic obstructive pulmonary disease rescue pack
**38074**	9Oi4.00	Chronic obstructive pulmonary disease monitor phone invite
**45777**	8CR1.00	Chronic obstructive pulmonary disease clini management plan
**19106**	66Yd.00	COPD accident and emergency attendance since last visit
**19003**	66Ye.00	Emergency COPD admission since last appointment
**42258**	9Oi3.00	Chronic obstructive pulmonary disease monitoring verb invite
**21061**	H3y0.00	Chronic obstruct pulmonary dis with acute lower resp infectn
**7092**	H30..12	Recurrent wheezy bronchitis
**103007**	66YB100	Chronic obstructive pulmonary disease 6 monthly review
**102685**	66YB000	Chronic obstructive pulmonary disease 3 monthly review
**19721**	8CE6.00	Chronic obstructive pulmonary disease leaflet given
**99948**	9kf0.00	COPD patient unsuitable for pulmonary rehab - enh serv admin
**104117**	661M300	COPD self-management plan agreed
**104169**	661N300	COPD self-management plan review
**46036**	66Yi.00	Multiple COPD emergency hospital admissions
**103494**	14B3.12	History of chronic obstructive pulmonary disease

© 2018 Lawson CA et al. *JAMA Network Open*.

**104265**	9e03.00	GP OOH service notified of COPD care plan
**103758**	8Hkw.00	Referral to COPD community nursing team
**103678**	8BMa000	Chronic obstructiv pulmonary disease medication optimisation
**45770**	66Yg.00	Chronic obstructive pulmonary disease disturbs sleep
**105457**	8CMW500	Chronic obstructive pulmonary disease care pathway
**97800**	9kf..00	COPD - enhanced services administration
**22905**	H581.00	Interstitial emphysema
**103558**	8CeD.00	Preferred place of care for next exacerbation of COPD
**104481**	8CMV.00	Has chronic obstructive pulmonary disease care plan
**98284**	9kf1.00	Refer COPD structured smoking assessment - enhanc serv admin
**65733**	Hyu3100	[X]Other specified chronic obstructive pulmonary disease
**98283**	9kf2.00	COPD structured smoking assessment declined - enh serv admin
**104985**	9NgP.00	On chronic obstructive pulmonary disease supprtv cre pathway
**103760**	9kf2.11	COPD structured smoking assessment declined
**103864**	9kf0.11	COPD patient unsuitable for pulmonary rehabilitation
**103400**	9kf1.11	Referred for COPD structured smoking assessment
**104710**	9NgP.11	On COPD (chr obstruc pulmonary disease) supportv cre pathway

© 2018 Lawson CA et al. *JAMA Network Open*.

eTable 3. Potential Confounders

Patient factors	Age<br>Gender<br>Index of Multiple Deprivation<br>Body mass index<br>Smoking<br>Alcohol
Clinical factors	Cholesterol<br>Haemoglobin<br>Blood pressure<br>Estimated glomerular filtration rate<br>Hypertension<br>Ischaemic heart disease<br>Myocardial infarction<br>Atrial fibrillation<br>Diabetes
Medications prescribed	Aspirin<br>Angiotensin converting enzyme inhibitors<br>Angiotensin II receptor blockers<br>Aldosterone antagonists<br>Beta-blockers<br>Diuretics

Index of Multiple Deprivation (IMD) measures deprivation linked to small housing areas in England and covering seven domains. Domain scores are combined using appropriate weights, generating a single overall IMD which is a useful proxy of individual level deprivation for use in health research. The score was ranked into quintiles, ranging from the lowest deprivation (quintile 1) to the highest (quintile 5).

English Indices of Deprivation 2010 available from: https://www.gov.uk/government/uploads/system/uploads/attachment_data/file/6871/1871208.pdf.

© 2018 Lawson CA et al. *JAMA Network Open*.

eTable 4. Missing Data Preimputation

	**Mortality sample**	**Hospitalisation subsample**
Patient characteristics	Missing No. (%)	Missing No. (%)
IMD	40.6	0.3
BMI (Kg/m2)	8.9	9.6
Cholesterol (mg/dL)	14.4	14.7
Hb (g/dL)	11.6	11.0
Systolic BP (mmHg)	0.6	0.5
Diastolic BP (mmHg)	0.6	0.5
eGFR	10.5	12.4
Smoking	2.4	2.4
Alcohol	10.1	10.1

IMD, index multiple deprivation; BMI, body mass index; Hb, haemoglobin; BP, blood pressure; eGFR, estimated glomerular filtration rate.

© 2018 Lawson CA et al. *JAMA Network Open*.

eTable 5. Characteristics of Patients With HF and COPD by Airflow Limitation in Those With Spirometry Data

	**Mortality sample**			**Hospitalisation subsample**		
**Patient characteristics**	**All (n=8,515)**	**GOLD 1&2[a] (n=5,005)**	**GOLD 3&4[a] (n=3,510)**	**All (n=4,652)**	**GOLD 1&2[a] (n=2,617)**	**GOLD 3&4[a] (n=2,035)**
Age, years; median [IQR]	78[IQR 72-83]	79[71-85]	77[71-82]	78[IQR 70-83]	79[71-84]	77[70-82]
Women; No. (%)	3,028(35.6)	1,910(38.2)	1,118(31.9)	1,778(38.4)	1,110(42.4)	678(33.3)
IMD quintile; No. (%)						
1	812(15.7)	461(15.0)	351(16.8)	710(15.3)	384(14.7)	326(16.0)
2	1,041(20.1)	630(20.4)	411(19.7)	915(19.7)	471(18.0)	444(22.8)
3	1,029(19.9)	632(20.5)	397(19.0)	910(19.6)	518(19.8)	392(19.3)
4	1,118(21.6)	642(20.8)	476(22.8)	1,056(22.7)	606(23.2)	450(22.1)
5	1,176(22.7)	719(23.3)	457(21.9)	1,061(22.8)	638(23.4)	423(20.8)
BMI (Kg/m2); median [IQR]	26.1[22.6-30.4]	26.6[23.0-30.8]	25.4[21.9-29.9]	26.3[22.9-30.5]	26.9[23.6-31.3]	25.5[22.0-29.0]
Cholesterol (mg/dL); mean (SD)	170.1±42.5	166.3±73.5	174±46.4	177.9±42.5	174.0±42.5	181.7±46.4
Hb (g/dL); mean (SD)	13.0±2.0	13.0±1.9	13.1±2.0	13.4±1.9	13.4±1.8	13.4±1.9
Systolic BP (mmHg); mean (SD)	126.8±19.9	126.8±19.6	126.9±20.3	131.4±19.6	131.8±19.4	130.9±19.9
Diastolic BP (mmHg); mean (SD)	70.9±11.4	70.9±11.2	71.0±11.6	73.0±11.5	73.2±11.6	72.7±11.4
Diuretics; No. (%)	6,987(82.1)	4,055(81.0)	2,932(83.5)	3,721(80.0)	2,033(77.7)	1,688(83.0)

© 2018 Lawson CA et al. *JAMA Network Open*.

Beta blocker; No. (%)	3,136(36.8)	2,071(41.4)	1,065(30.3)	961(20.7)	640(24.5)	321(15.8)
ACEi; No. (%)	4,612(54.2)	2,719(54.3)	1,893(53.9)	2,774(59.6)	1,553(59.3)	1,221(60.0)
ARB; No. (%)	1,439(16.9)	4,051(19.1)	3,025(86.2)	699(15.0)	460(17.6)	239(11.7)
Aspirin; No. (%)	6,382(75.0)	3,889(77.7)	2,493(77.7)	3,185(68.5)	1,875(71.7)	1,310(64.4)
AA; No. (%)	1,847(21.7)	1,119(22.4)	728(71.0)	578(12.4)	326(12.5)	252(12.4)
COPD medications; No. (%)						
No medications	935(11.0)	689(13.8)	246(7.0)	741(15.9)	464(17.7)	277(13.6)
Short term inhalers only	632(7.4)	435(8.7)	197(5.6)	427(9.2)	293(11.2)	134(6.7)
Monotherapy	1,020(12.0)	699(14.0)	321(9.2)	576(12.4)	367(14.0)	209(10.3)
Dual therapy	1,545(18.1)	996(19.9)	549(15.6)	894(19.2)	576(22.0)	318(15.6)
Triple therapy	1,639(19.30	862(17.2)	777(22.1)	764(16.4)	336(12.8)	428(21.0)
Oral steroids but no oxygen	2,385(28.0)	1,185(23.7)	1,198(34.1)	1,152(24.8)	554(21.2)	598(29.3)
Oxygen therapy	361(4.2)	139(2.8)	222(6.3)	98(2.1)	27(1.0)	71(3.5)
Diabetes; No. (%)	2,018(23.7)	1,276(25.5)	742(21.1)	827(17.8)	504(19.3)	323(15.9)
Renal disease (eGFR <60 ml/min/m^2); No. (%)	4,039(50.3)	2,548(54.3)	1,455(44.5)	1,551(47.5)	945(50.8)	606(43.2)
Atrial fibrillation ; No. (%)	3,215(37.8)	1,956(32.7)	1,259(35.9)	1,493(32.1)	851(32.5)	642(31.6)
Hypertension; No. (%)	4,703(55.2)	2,914(58.2)	1,789(51.0)	2,434(52.3)	1,486(56.8)	948(46.6)
Ischamic heart disease; No. (%)	3,604(42.3)	2,318(46.3)	1,286(36.6)	1,815(39.0)	1,126(43.0)	689(33.9)
Myocardial infarction; No. (%)	2,315(27.2)	1,457(29.1)	2,414(24.4)	875(18.8)	582(22.2)	293(14.2)

© 2018 Lawson CA et al. *JAMA Network Open*.

Smoking status; No. (%)						
Current	1,242(14.6)	766(15.3)	476(13.6)	915(19.7)	468(17.9)	447(22.0)
Not current	5,632(66.1)	3,328(66.5)	2,304(65.6)	661(14.2)	380(14.5)	281(13.8)
Past smoker	1,641(19.3)	911(18.2)	730(20.8)	3,076(66.1)	1,769(67.6)	1,307(64.2)
Alcohol status; No. (%)						
Current	1,979(23.2)	1,139(22.8)	840(23.9)	3,481(74.8)	1,964(75.1)	1,517(74.6)
Not current	528(6.2)	305(6.1)	223(6.4)	980(21.1)	562(21.5)	418(20.5)
Past drinker	6,008(70.6)	3,561(71.2)	2,447(69.7)	191(4.1)	91(3.5)	100(4.9)

HF, heart failure; IMD, index multiple deprivation (1=least deprived, 5=most deprived); BMI, body mass index; Hb, haemoglobin; BP, blood pressure; ACEi, angiotensin-converting enzyme inhibitor; ARB, angiotensin receptor blocker; AA, aldosterone antagonist (spironolactone or eplerenone); COPD, chronic obstructive pulmonary disease; eGFR, estimated glomerular filtration rate

[a]GOLD stages refer to airflow limitation grades in GOLD guidelines. 1: FEV1 ≥80% normal (mild) 2: FEV1 50-79% normal (moderate) 3: FEV1 30-49% normal (severe) 4: FEV1 <30% normal (very severe).

© 2018 Lawson CA et al. *JAMA Network Open*.

eTable 6. Characteristics of Patients With HF and COPD by Spirometry Availability

Patient characteristics	Mortality sample		Hospitalisation subsample	
	COPD patients without spirometry (n=9,693)	COPD patients with spirometry (n=8,515)	COPD patients without spirometry (n=7,251)	COPD patients with spirometry (n=4,652)
Age, years; median [IQR]	77[IQR 70-83]	78[IQR 72-83]	76[IQR 69-82]	78[IQR 70-83]
Women; No. (%)	4,219(42.4)	3,028(35.6)	3,194(44.31)	1,778(38.4)
IMD quintile; No. (%)				
1	852(14.5)	812(15.7)	1,077(14.9)	710(15.3)
2	1,196(20.3)	1,041(20.1)	1,499(20.0)	915(19.7)
3	1,262(21.4)	1,029(19.9)	1,551(21.4)	910(19.6)
4	1,402(23.8)	1,118(21.6)	1,661(22.9)	1,056(22.7)
5	1,185(20.1)	1,176(22.7)	1,514(20.9)	1,061(22.8)
BMI (Kg/m2); median [IQR]	27.1[23.5-31.6]	26.1[22.6-30.4]	27.1[23.8-31.8]	26.3[22.9-30.5]
Cholesterol (mg/dL); mean (SD)	177.9±46.4	170.1±42.5	185.6±46.4	177.9±42.5
Hb (g/dL); mean (SD)	13.2±1.8	13.0±2.0	13.6±1.7	13.4±1.9
Systolic BP (mmHg); mean (SD)	130.7±19.7	126.8±19.9	133.6±19.9	131.4±19.6
Diastolic BP (mmHg); mean (SD)	73.1±11.0	70.9±11.4	75.1±11.0	73.0±11.5
Diuretics; No. (%)	7,970(80.0)	6,987(82.1)	5,304(73.2)	3,721(80.0)
Beta blocker; No. (%)	3,650(36.6)	3,136(36.8)	1,430(19.7)	961(20.7)
ACEi; No. (%)	5,348(53.7)	4,612(54.2)	4,141(57.1)	2,774(59.6)
ARB; No. (%)	1,597(16.0)	1,439(16.9)	1,193(16.5)	699(15.0)
Aspirin; No. (%)	7,149(71.9)	6,382(75.0)	4,696(64.8)	3,185(68.5)
AA; No. (%)	2,019(20.3)	1,847(21.7)	1,002(13.8)	578(12.4)
Diabetes; No. (%)	2,258(22.7)	2,018(23.7)	1,280(17.7)	827(17.8)
Renal disease (eGFR <60 ml/min/m^2); No. (%)	4,435(49.5)	4,039(50.3)	3,265 (45.0)	1,551(47.5)
Atrial fibrillation ; No. (%)	3,261(32.7)	3,215(37.8)	2,006(27.7)	1,493(32.1)
Hypertension; No. (%)	5,393(54.1)	4,703(55.2)	3,754(51.8)	2,434(52.3)
Ischamic heart disease; No. (%)	3,980(40.0)	3,604(42.3)	2,502(34.5)	1,815(39.0)
Myocardial infarction; No. (%)	2,414(24.2)	2,315(27.2)	1,360(18.8)	875(18.8)
Smoking status; No. (%)				
Current	1,950(19.6)	1,242(14.6)	1,606(22.2)	915(19.7)
Not current	5,836(58.6)	5,632(66.1)	1,406(19.4)	661(14.2)

© 2018 Lawson CA et al. *JAMA Network Open*.

Past smoker	2,177(21.9)	1,641(19.3)	4,239(58.5)	3,076(66.1)
Alcohol status; No. (%)				
Current	2,526(25.4)	1,979(23.2)	5,250(72.4)	3,481(74.8)
Not current	577(5.8)	528(6.2)	1,633(22.5)	980(21.1)
Past drinker	6,860(68.9)	6,008(70.6)	633(5.1)	191(4.1)

HF, heart failure; IMD, index multiple deprivation (1=least deprived, 5=most deprived); BMI, body mass index; Hb, haemoglobin; BP, blood pressure; ACEi, angiotensin-converting enzyme inhibitor; ARB, angiotensin receptor blocker; AA, aldosterone antagonist (spironolactone or eplerenone); COPD, chronic obstructive pulmonary disease; eGFR, estimated glomerular filtration rate.

© 2018 Lawson CA et al. *JAMA Network Open*.

eTable 7. Interactions

	Mortality sample		Hospitalisation subsample	
	Adjusted OR (95% CI)[a]	Interaction term (P)	Adjusted OR (95% CI)[a]	Interaction term (P)
HF with no COPD (reference group)	1		1	
COPD in females	1.41(1.30,1.53)		1.37(1.25,1.50)	
COPD in males	1.26(1.18,1.34)	0.007	1.31(1.21,1.42)	0.748
COPD in those prescribed beta blockers	1.40(1.29,1.52)		1.25(1.09,1.43)	
COPD in those not prescribed beta blockers	1.22(1.15,1.31)	0.06	1.35(1.26,1.43)	0.09

COPD, chronic obstructive pulmonary disease; OR, odds ratio; CI confidence interval.

[a] All models adjusted for age, gender, body mass index (BMI), cholesterol, estimated glomerular filtration rate (eGFR), eGFR2, Haemoglobin (Hb), Hb2, systolic blood pressure (SBP), SBP2, diuretics, beta-blocker, angiotensin-converting enzyme inhibitor; angiotensin receptor blocker; aldosterone antagonist (spironolactone or eplerenone), Aspirin, atrial fibrillation, hypertension, ischemic heart disease, myocardial infarction, diabetes, smoking, alcohol and previous hospitalisation in 12-months (Hospitalisation models only).

© 2018 Lawson CA et al. *JAMA Network Open*.

eTable 8. Sensitivity Analyses

	**Mortality**	**Hospitalisation**
	Adjusted OR (95% CI)[a]	Adjusted OR (95% CI)[a]
HF with no COPD (reference group)	1	1
COPD	1.31(1.26,1.36)	1.32(1.24,1.42)
COPD adjusted additionally for deprivation	1.28(1.20,1.36)	N/A
COPD in those with a spirometry recording	1.93(1.83,2.04)	1.53(1.42,1.65)
COPD in those without a spirometry recording	0.87(0.82,0.92)[b]	1.20(1.12,1.26) [b]
COPD: HF index before 1st April 2006	1.36(1.28,1.44)	1.39(1.29,1.50)
COPD: HF index after 1st April 2006	1.27(1.19,1.35)[b]	1.28(1.19,1.36)[b]
COPD in those without spirometry(reference group)	1	1
COPD in those with a spirometry recording	2.22(2.06,2.39)	1.28(1.17,1.40)

COPD, chronic obstructive pulmonary disease; OR, odds ratio; CI confidence interval.

[a]All models adjusted for age, gender, body mass index (BMI), cholesterol, estimated glomerular filtration rate (eGFR), eGFR2, Haemoglobin (Hb), Hb2, systolic blood pressure (SBP), SBP2, diuretics, beta-blocker, angiotensin-converting enzyme inhibitor; angiotensin receptor blocker; aldosterone antagonist (spironolactone or eplerenone), Aspirin, atrial fibrillation, hypertension, ischemic heart disease, myocardial infarction, diabetes, smoking, alcohol and previous hospitalisation in 12-months (Hospitalisation models only).

[b]Significant interaction (P>0.05)

© 2018 Lawson CA et al. *JAMA Network Open*.
